# Accelerated DNA methylation age plays a role in the impact of cardiovascular risk factors on the human heart

**DOI:** 10.1186/s13148-023-01576-9

**Published:** 2023-10-18

**Authors:** Constantin-Cristian Topriceanu, Eesha Dev, Mahmood Ahmad, Rebecca Hughes, Hunain Shiwani, Matthew Webber, Kenan Direk, Andrew Wong, Martin Ugander, James C. Moon, Alun D. Hughes, Jane Maddock, Todd T. Schlegel, Gabriella Captur

**Affiliations:** 1 UCL MRC Unit for Lifelong Health and Ageing, University College London, 1-19 Torrington Place, London, UK; 2 UCL Institute of Cardiovascular Science, University College London, 62 Huntley St, London, WC1E 6BT UK; 3 Cardiac MRI Unit, Barts Heart Centre, West Smithfield, London, UK; 4 UCL Medical School, Gower Street, London, UK; 5 Centre for Inherited Heart Muscle Conditions, The Royal Free Hospital, Pond Street, Hampstead, London, UK; 6 Kolling Institute Royal North Shore Hospital, and Charles Perkins Centre, Faculty of Medicine and Health, University of Sydney, Sydney, Australia; 7 Department of Clinical Physiology, Karolinska University Hospital, and Karolinska Institutet, Stockholm, Sweden; 8 Nicollier-Schlegel SARL, Trélex, Switzerland

**Keywords:** Advanced electrocardiography, Aging, DNA methylation age

## Abstract

**Background:**

DNA methylation (DNAm) age acceleration (AgeAccel) and cardiac age by 12-lead advanced electrocardiography (A-ECG) are promising biomarkers of biological and cardiac aging, respectively. We aimed to explore the relationships between DNAm age and A-ECG heart age and to understand the extent to which DNAm AgeAccel relates to cardiovascular (CV) risk factors in a British birth cohort from 1946.

**Results:**

We studied four DNAm ages (AgeHannum, AgeHorvath, PhenoAge, and GrimAge) and their corresponding AgeAccel. Outcomes were the results from two publicly available ECG-based cardiac age scores: the Bayesian A-ECG-based heart age score of Lindow *et al. 2022* and the deep neural network (DNN) ECG-based heart age score of Ribeiro *et al. 2020.* DNAm AgeAccel was also studied relative to results from two logistic regression-based A-ECG disease scores, one for left ventricular (LV) systolic dysfunction (LVSD), and one for LV electrical remodeling (LVER). Generalized linear models were used to explore the extent to which any associations between biological cardiometabolic risk factors (body mass index, hypertension, diabetes, high cholesterol, previous cardiovascular disease [CVD], and any CV risk factor) and the ECG-based outcomes are mediated by DNAm AgeAccel. We derived the total effects, average causal mediation effects (ACMEs), average direct effects (ADEs), and the proportion mediated [PM] with their 95% confidence intervals [CIs]. 498 participants (all 60–64 years) were included, with the youngest ECG heart age being 27 and the oldest 90. When exploring the associations between cardiometabolic risk factors and Bayesian A-ECG cardiac age, AgeAccelPheno appears to be a partial mediator, as ACME was 0.23 years [0.01, 0.52] *p* = 0.028 (i.e., PM≈18%) for diabetes, 0.34 [0.03, 0.74] *p* = 0.024 (i.e., PM≈15%) for high cholesterol, and 0.34 [0.03, 0.74] *p* = 0.024 (PM≈15%) for any CV risk factor. Similarly, AgeAccelGrim mediates ≈30% of the relationship between diabetes or high cholesterol and the DNN ECG-based heart age. When exploring the link between cardiometabolic risk factors and the A-ECG-based LVSD and LVER scores, it appears that AgeAccelPheno or AgeAccelGrim mediate 10–40% of these associations.

**Conclusion:**

By the age of 60, participants with accelerated DNA methylation appear to have older, weaker, and more electrically impaired hearts. We show that the harmful effects of CV risk factors on cardiac age and health, appear to be partially mediated by DNAm AgeAccelPheno and AgeAccelGrim. This highlights the need to further investigate the potential cardioprotective effects of selective DNA methyltransferases modulators.

**Supplementary Information:**

The online version contains supplementary material available at 10.1186/s13148-023-01576-9.

## Introduction

The biological aging process is complex and involves a range of cellular and molecular changes that accumulate over time, resulting in deterioration of physiological function and increased vulnerability to chronic diseases, and eventually increased mortality [1]. Heterogeneity across human biological aging phenotypes cannot be solely explained by chronological age [2]. Therefore, biological aging biomarkers capable of identifying individuals at risk of functional impairment beyond chronological age have been extensively researched.

The most promising biological aging biomarkers are those based on DNA methylation (DNAm) levels at specific cytosine-phosphate-guanine (CpG) sites [3]. The 1st generation DNAm ages were created using ElasticNet penalized regression in which CpG sites were used to predict chronological age. DNAm AgeHannum [4] was based on blood cells, whilst DNAm AgeHorvath [5] incorporated methylation data from multiple tissues. Both were highly correlated with chronological age but poorly associated with CpG sites that captured lifespan and healthspan. As a result, 2nd generation DNAm biomarkers emerged: DNAm PhenoAge based on whole-blood CpG sites which associated with a composite of mortality-related clinical and physiological measures [6]; and DNAm GrimAge based on plasma protein estimates, smoking pack-years, chronological age and sex as a function of the time-to-death [7]. After regressing these DNAm ages on chronological age, the residuals can be interpreted as a measure of age acceleration (AgeAccel), providing valuable insights into the speed of the epigenetic clock. A positive residual value is adverse as it suggests faster biological aging, while a negative value is beneficial as it suggests slower biological aging. The 2nd generation AgeAccel DNAm biomarkers have been shown to be more predictive of adverse health outcomes than their 1st generation DNAm counterparts [8, 9].

Cardiac ageing is naturally associated with a progressively increasing burden of cardiovascular diseases (CVD) across the human lifespan. Given the complexity of cardiac aging, chronological age is not an optimal proxy [10]. Thus, cardiac ages based on imaging, electrocardiography (ECG) or cardiometabolic data have been proposed [11, 12]. Of these, advanced electrocardiography (A-ECG) which incorporates results from conventional ECG, vectorcardiography and waveform complexity within Bayesian statistical frameworks, or alternatively within  deep neural network (DNN) ECG-based algorithms, has the highest translational potential given the low cost and the ubiquitous availability of ECG across healthcare systems [13–15].

Underpinning both DNAm and cardiac aging are a set of shared risk factors such as diet [16], smoking, exercise [16], lifetime psychological stress [17] and ambient air pollution [18], amongst others. Since many of these risk factors were shown to have different epigenetic methylation signatures at specific CpG sites [19, 20], the existence of a strong link between DNAm and cardiac aging was previously postulated [21]. Indeed, although 1st generation DNAm ages appear be weakly linked to CVDs [22], the 2nd generation AgeAccel DNAm were shown to be more predictive of adverse cardiovascular outcomes [8, 9]. As distinct DNAm profiles have been associated with cardiometabolic risk factors (e.g., body mass index [BMI] [23], diabetes [24], high cholesterol [25], hypertension [26] and coronary artery disease [27]), the idea that DNAm might be a mediator between CV risk factors and cardiac age gained traction. Thus, the role of DNA methyltransferase inhibitors (DNMT-i) was studied in pre-clinical trials with encouraging effects in ameliorating cardiac hypertrophy [28], fibrosis [29] and atherosclerosis [30]. However, to what extent AgeAccel DNAm might mediate the effect of biological CV risk factors on ECG-based cardiac age remains to be elucidated. Using prospectively collected life-course data from the 1946 Medical Research Council (MRC) British National Survey of Health and Development (NSHD) study, we sought to answer this question.

## Methods

### Study population

The MRC NSHD is a birth cohort study that includes 5,362 individuals (2,547 males and 2,815 females) who were born in one week in March 1946 in Britain. This cohort has been extensively followed up with periodic assessments of various aspects of their lives, including anthropometric measurements, socio-economic status, lifestyle factors, and health outcomes [31].

### Advanced electrocardiography

Between 2006 and 2010, when NSHD participants were 60–64 years old, those residing in the UK who had not been lost to follow-up or withdrawn were invited to attend a clinic-based assessment which included a standard 12-lead surface ECG. The 10-sec ECGs were stored in digital format to avoid the signal quality degradation that affects paper traces.

Based on the earlier method of Ball *et al.* [32], Lindow *et al.* [15] recently implemented a machine learning-based Bayesian-centric approach to predict cardiac age from multiple discrete features derivable from standard 12-lead ECGs, combining inputs from: (1) conventional ECG durations (e.g., P and QTc), amplitudes and axes (e.g., QRS and T); (2) the spatial QRS-T angles, spatial ventricular gradient, spatial QRS- and T-wave axes, azimuths, elevations, velocities, waveform amplitudes and areas from the derived, Frank X,Y, and Z lead vectorcardiogram; and (3) QRS- and T-waveform complexity obtained via singular value decomposition after signal averaging. Univariable linear regression models were used to select the ECG features, and multivariable linear regression models to estimate the cardiac age from the original Bayesian A-ECG heart age model of Ball *et al.* that had used higher-fidelity 5-min A-ECGs [32]. The model of Lindow *et al. *[15] was utilized in the present study to derive the estimated Bayesian A-ECG cardiac age for each member of our own cohort from their respective, standard 12-lead ECGs.

The results from two logistic regression-based A-ECG scores for cardiac diseases were also evaluated: one for left ventricular (LV) systolic dysfunction (LVSD), and one for LV electrical remodeling (LVER) [33–38]. Both the LVSD and the LVER A-ECG scores prominently incorporate results from the spatial QRS-T angle, a measure also known to have important prognostic utility [39]. While the results from these scores comprise continuous variables (as utilized in this study) rather than categorical variables, the presence of the given disease by A-ECG is usually also clinically defined as the score’s related probability exceeding 0.5 (50%). The presence of a positive LVSD score was originally designed to correspond to an imaging-proven left ventricular ejection fraction (LVEF) < 50% [33]. However, ongoing research and subsequent clinical practice suggest that the LVSD score slightly better correlates with changes in global longitudinal strain than in LVEF [34, 35]. The presence of a positive LVER score was in turn designed to more accurately predict (versus strictly conventional ECG criteria) the presence of moderate or greater left ventricular hypertrophy by gold-standard imaging [33, 37, 38].

Finally, among others, Ribeiro *et al*. [13, 14] have also recently designed a deep neural network (DNN) to estimate the cardiac age based on raw, standard 12-lead ECG tracings. The DNN of Ribeiro *et al.* consists of 11 convolutional layers (with the last 10 organized into 5 blocks) whose weights were initialized via random sampling from a scaled normal variable. It was trained on a dataset of 1,558,415 patients from 811 counties in the state of Minas Gerais (Brazil) collected by the Telehealth Network of Minas Gerais (TNMG). Batch normalization was employed to rescale the output of each convolutional layer before being fed into a rectified linear activation unit, with an Adam optimizer being used to minimize the mean square error. Ribeiro *et al.* have made their DNN model publicly available [13, 14]. Thus DNN ECG-based cardiac ages were also derived for our own cohort by using Ribeiro *et al.*’s publicly accessible algorithm.

### DNA methylation-based aging biomarkers

Blood samples from NSHD study members were collected in 1999 and again between 2006 and 2010 as previously described [31]. Illumina Infinium Methylation EPIC BeadChips kits (Illumina, San Diego, California, US) were used to measure DNAm signals at > 850,000 CPG sites. The signals were processed for quality control (QC) in the R Enmix package [40], and beta-values were obtained using the noob normalization method in the R minfi package [41]. Signals with a detection *p*-value > 10^–6^ and a number of beads < 3 were set to missing. We excluded: (1) samples with missing data in > 5% of the CpGs; (2) CpGs with missing data in > 5% of the samples; and (3) samples with outliers in bisulfite intensity, total intensity, or beta-values. Outliers were defined as values more than 3 standard deviations (SDs) from the mean or 3 interquartile ranges (IQRs) below the 1st or above the 3rd quartiles. Sample identity was verified by calculating the Pearson correlation coefficient between the 59 methylation bead chips SNPs and the imputed genotype data yielding coefficients > 0.90.

We calculated DNAm AgeHannum, DNAm AgeHorvath, DNAm PhenoAge and DNAm GrimAge using the methodology described by Horvath which is also available as online age calculator (https://dnamage.genetics.ucla.edu/home) [5]. Age acceleration (in years) was defined as the residual produced by linearly regressing DNAm age on the chronological age [42], yielding the corresponding AgeAccelHannum, AgeAccelHorvath, AgeAccelPheno and AgeAccelGrim.

### Cardiometabolic risk factors and covariates

Sex was assigned at birth as male or female. During the same clinic visit when ECG was recorded, participants’ weight and height were also measured and used to compute body mass index (BMI). Participants’ socioeconomic position (SEP) was evaluated at the time of echocardiography (60–64 years) or at 53 years where the former was not available, according to the UK Office of Population Censuses and Surveys Registrar General’s occupational-based social class dichotomized as manual or non-manual. Self-reported questionnaires at 60–64 years also provided information about smoking status (never smoked, ex-smoker and currently smoking), the average number of units of alcohol consumed per day and leisure time physical activity. The latter was dichotomized as inactive or active (exercises at least once per month). Blood samples at 60–64 were analyzed to provide the white cell counts: naïve and exhausted CD8+ T-lymphocytes, CD4+ T-lymphocytes, B-cells, natural killer cells, granulocytes, and monocytes. The presence of CVD, diabetes, high cholesterol or hypertension was recorded as 1 = present or 0 = absent as previously described [43]. In addition, we defined the presence of ‘any CV risk factor’ as any participant having at least one out of diabetes, high cholesterol, hypertension, CVD, or a BMI > 30.

### Statistics

Statistical analysis was performed in R (version-4.2.1), and a two-tailed *p* value < 0.05 was considered statistically significant. Distributions of data were assessed on histograms and using the Shapiro–Wilk test. Continuous variables were expressed as mean ± 1 SD or median (IQR) as appropriate; and categorical variables, as counts and percentages.

In all analyses, the DNAm age or AgeAccel were the independent variables, whilst the A-ECG-based and DNN ECG-based cardiac ages or the A-ECG-based LVSD and LVER scores were the outcomes. To test for associations, we employed generalized linear models (glms) with Gaussian distributions and identity links. To mitigate the influence of confounders, the models were adjusted for chronological age, sex, SEP, smoking, alcohol consumption, and physical activity. To obtain better estimates of the actual epigenetic changes, the models were also adjusted for the white cell counts. We repeated the analyses for each DNAm age (i.e., AgeHannum, AgeHorvath, PhenoAge and GrimAge) as well as for the corresponding AgeAccel.

**Fig. 1:**
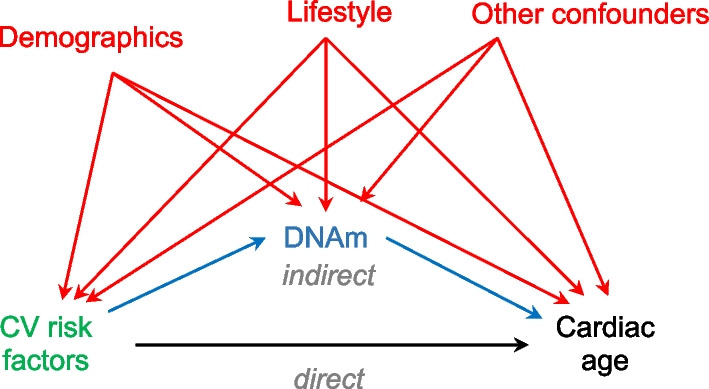
**Directed acyclic graph highlighting the assumed potential causal relationships between CV risk factors, DNAm and cardiac age**. The exposure is shown in green, mediator in blue, outcome in black, and confounders in red. The presence of CV risk factors (e.g., BMI, diabetes, hypertension, high cholesterol, and previous CVD) was associated with higher ECG-based cardiac ages. However, this relationship could be confounded by demographics (e.g., age, sex, and socio-economic position) and lifestyle varibles (e.g., physical activity, smoking and alcohol consumption), amongst others. After adjusting for the confounders and for the white cell counts, the mediator analysis identified indirect effects on ECG-based cardiac ages through DNAm AgeAccelPheno and AgeAccelGrim especially for diabetes, high cholesterol, and hypertension, accounting in general for < 40% of the total effects. This highlights the potential role of DNAm as a mediator, downstream of CV risk factors but upstream of cardiac age. Abbreviations: AgeAccel = age acceleration; ECG = electrocardiography; BMI = body mass index; CV = cardiovascular; CVD = cardiovascular diseases; DNAm = DNA methylation

Firstly, we explored the associations between the DNAm ages and the A-ECG and DNN ECG-related outcomes. Secondly, we explored to what extent DNAm AgeAccel mediates the effect of CV risk factors (BMI, diabetes, high cholesterol, hypertension, CVD and ‘any CV risk factor’) on the A-ECG and DNN ECG-related outcomes using mediation analysis. The hypothetical mediation mechanism is presented in Fig. 1. We used the counterfactual framework methodology of causal inference developed by Imai, Tingley and Yamamoto which relies on the no-interaction (i.e., no exposure-mediator and mediator-outcome interactions) and sequential ignorability (SI; i.e., the absence of unmeasured confounding) assumptions [44–46]. To calculate the total effects (the direct model), we regressed the CV risk factors on the A-ECG and DNN ECG-related outcomes. To calculate the effect of the independent variable onto the mediator (the mediator model), we regressed the CV risk factors on the DNAm AgeAccel. To calculate the effect of the mediator on the dependent variable (the outcome model), we regressed the DNAm AgeAccel on the A-ECG and DNN ECG-related outcomes whilst adjusting for the CV risk factors. Then, we derived the total effects, average causal mediation effects (ACMEs) and average direct effects (ADEs) with their 95% confidence intervals (CIs) using nonparametric bootstrapping with 1000 Monte Carlo simulations [47]. The proportion mediated (PM) was derived by dividing ACME by the total effect for each analysis. To investigate the robustness of our results, we used the Baron-Kenny procedure to calculate the correlation coefficients (ρ) between the residuals of the mediator and the regression outcomes from the linear structural equation models (LSEM) rather than glms as this was the only available implementation in R. We judged the robustness of the results by the magnitude of ρ required to reverse the sign of ACME.

## Results

Participant characteristics for the whole cohort, along with those stratified by sex, are showcased in Table 1. On average, male participants were more likely to have a non-manual-labor SEP (34.9% vs. 26.0%, *p* = 0.039), high cholesterol (29.0% vs. 13.4%, *p* < 0.0001), hypertension (57.5% vs. 43.9%, *p* = 0.003) and CVD (10.7% vs. 3.3%, *p* = 0.005). Conversely, females were more likely to have a lower DNAm AgeHannum (49.9 vs. 52.9, *p* < 0.0001), DNAm AgeHorvath (57.3 vs. 58.7, *p* < 0.0001), DNAm GrimAge (60.0 vs. 63.0, *p* < 0.0001) and hence a higher AgeAccel in these DNAm ages. Males were more likely to have a worse DNN ECG-related cardiac ages (62.7 vs. 59.6, *p* = 0.005), and females a worse Bayesian A-ECG cardiac age (70.2 vs. 68.0, *p* < 0.0001). Males had a worse LVSD score, whilst the LVER score was not statistically different based on sex.

**Table 1:** Participant characteristics

	Overall (*n* = 498)	Women (*n* = 246)	Men (*n* = 252)	*p* value
Demographics				
Age, years	63.48 (62.75, 63.97)	63.47 (60.48, 63.95)	63.49 (62.45, 63.97)	0.710
SEP, Non-Manual	152 (30.5%)	64 (26.0%)	88 (34.9%)	**0.039**
Smoking				
Current smoker	47 (10.1%)	26 (5.6%)	21 (4.5%)	**1.2e−05**
Ex-smoker	190 (40.7%)	68 (14.6%)	122 (26.1%)	
Never smoked	230 (49.2%)	135 (28.9%)	95 (20.3%)	
Alcohol, units/day	3.4 (2.1, 4.02)	2.7 (1.9, 4.4)	4.0 (2.7, 6.0)	**1.24e−06**
Physical activity, yes (exercise ≥ 1 time/month)	188 (42.7%)	96 (43.7%)	92 (41.8%)	0.923
Cardiometabolic exposures				
BMI, kg/m^2^	27.20 (24.62, 30.20)	26.81 (24.52, 30.73)	27.46 (24.94, 30.00)	0.338
Diabetes, yes	107 (21.5%)	45 (18.3%)	62 (24.6%)	0.138
High cholesterol, yes	106 (21.3%)	33 (13.4%)	73 (29.0%)	**3.7e−05**
Hypertension, yes	253 (50.8%)	108 (43.9%)	145 (57.5%)	**0.003**
CVD, yes	35 (7.0%)	8 (3.3%)	27 (10.7%)	**0.002**
Any CV risk factor, yes	340 (68.3%)	153 (62.2%)	187 (74.2%)	**0.005**
DNAm mediators				
DNAm AgeHannum, years	51.70 (48.94, 54.28)	49.91 (47.87, 52.61)	52.88 (50.71, 55.46)	**2.4e−16**
DNAm AgeHorvath, years	57.91 (55.47, 60.46)	57.30 (54.85, 59.56)	58.69 (56.26, 61.26)	**5.6e−05**
DNAm PhenoAge, years	48.03 (44.42, 51.88)	47.82 (43.75, 51.98)	48.14 (44.85, 51.74)	0.216
DNAm GrimAge, years	61.70 (59.36, 65.23)	60.02 (57.95, 63.24)	63.04 (61.08, 66.82)	**2.2e−16**
AgeAccelHannum, years	− 0.21 (− 2.99, 2.31)	− 2.14 (− 4.13, 0.72)	1.19 (− 1.20, 3.60)	**<2e−16**
AgeAccelHorvath, years	− 0.21 (− 2.65, 2.57)	− 0.79 (− 3.3, 1.55)	0.62 (− 2.01, 3.04)	**1e−04**
AgeAccelPheno, years	− 0.55 (− 4.05, 3.47)	− 0.91 (− 4.85, 3.63	0.08 (− 3.57, 3.38)	0.118
AgeAccelGrim, years	− 1.18 (− 3.46, 2.39)	− 2.66 (− 4.85, 0.02)	0.15 (− 1.96, 3.79)	**3.1e−16**
A-ECG outcomes				
Bayesian A-ECG age, years	68.98 (65.66, 72.39)	70.21 (66.64, 73.37)	67.95 (65.05, 71.14)	**2.5e−06**
DNN ECG-based age, years	60.59 (52.91, 68.67)	59.62 (51.70, 66.39)	62.65 (54.68, 69.71)	**0.005**
LVSD	2.59 (− 1.67, 3.59)	2.21 (1.45, 3.12)	3.04 (2.04, 4.06)	**1.8e−07**
LVER	3.66 (− 0.21, 6.63)	3.63 (− 0.34, 6.28)	3.68 (− 0.18, 7.19)	0.497

All variables are presented as counts (percentages) if categorial or median (interquartile range) if continuous. Comparisons were made using the Chi-Squared test with Yates continuity correction for categorical and Mann–Whitney U-test for continuous variables. Significant p-values are presented in bold

A-ECG = advanced electrocardiography; AgeAccel = age acceleration; BMI = body mass index; CV = cardiovascular, CVD = cardiovascular disease; DNAm = DNA methylation; DNN = deep neural network; LVER = left ventricular electrical remodeling; LVSD = left ventricular systolic dysfunction; SEP = socio-economic position

### Associations between DNAm ages and ECG-based cardiac ages and disease scores

The DNAm AgeHannum, AgeHorvath, PhenoAge and GrimAge were weakly correlated with both the Bayesian A-ECG and DNN ECG-based ages as well as with the LVSD and LVER disease scores (Table 2). A 1-year increase in the DNAm AgeHorvath or PhenoAge was associated with a ≈ 0.1 years (both *p* < 0.023) increase in the Bayesian A-ECG cardiac age, whilst a 1-year increase in DNAm GrimAge was associated with 0.21 years ([0.05, 0.37], *p* = 0.009) increase. Similarly, a 1-year increase in the DNAm AgeHorvath, PhenoAge or GrimAge was associated with a 0.3, 0.2, and 0.4 years respectively, increase in the DNN-ECG-based age. A higher DNAm AgeHorvath, PhenoAge and GrimAge were associated with worse LVSD and LVER scores.

**Table 2:** Associations between the DNAm ages and ECG-based cardiac ages and disease scores

	DNAm AgeHannum			DNAm AgeHorvath			DNAm PhenoAge			DNAm GrimAge		
	Correlation	Association		Correlation	Association		Correlation	Association		Correlation	Association	
	*ρ*	*β* (95% CI)	*p* value	*ρ*	*β* (95% CI)	*p* value	*ρ*	*β* (95% CI)	*p* value	*ρ*	*β* (95% CI)	*p* value
Bayesian A-ECG age	− 0.03	0.01 (− 0.10, 0.12)	0.875	0.08	0.09 (− 0.02, 0.21)	**0.023**	0.13	0.13 (0.05, 0.22)	**0.003**	0.06	0.21 (0.05, 0.37)	**0.009**
DNN ECG-based age	0.12	0.16 (− 0.09, 0.40)	0.462	0.10	0.27 (0.02, 0.52)	**0.033**	0.11	0.21 (0.02, 0.39)	**0.035**	0.16	0.44 (0.09, 0.79)	**0.014**
LVSD score	0.01	− 0,02 (− 0.06, 0.01)	0.204	− 0.03	− 0.03 (− 0.07, 0.01)	0.130	− 0.12	− 0.03 (− 0.06, − 0.01)	**0.022**	− 0.05	− 0.04 (− 0.10, 0.01)	0.127
LVER score	− 0.08	− 0.13 (− 0.26, 0.01)	0.065	− 0.08	− 0.14 (− 0.28, − 0.01)	**0.041**	− 0.13	− 0.15 (− 0.26, − 0.05)	**0.004**	− 0.10	− 0.21 (− 0.40, − 0.01)	**0.036**

Correlation was evaluated using Pearson’s correlation coefficients, while the association was appraised using generalized linear models with Gaussian distributions and identity links using DNAm ages as the independent variables and the -ECG-based cardiac ages or disease scores as the dependent variables. To obtain better estimates, the association models were adjusted for chronological age, sex, aSEP (as a binary variable: manual and non-manual), smoking status (as current smoker, ex-smoker and never-smoked), alcohol consumption (as the average number of alcohol units drank per day), physical activity status (as active or inactive) and white cell counts (i.e., naïve and exhausted CD8 + T-lymphocytes, CD4 + T-lymphocytes, B-cells, natural killer cells, granulocytes, and monocytes). Significant *p* values are in bold

*β* = regression coefficient; ρ = Pearson's correlation coefficient; CI = confidence interval. Other abbreviations as in Table 1

### DNAm as a mediator downstream of CV risk factors and upstream of ECG-based cardiac ages

Per Table 3, a 1-unit increase in BMI resulted in 0.32 years ([0.21, 0.44], *p* < 0.001) increase in the Bayesian A-ECG cardiac age. Moreover, having diabetes, high cholesterol, hypertension, or any CV risk factor, resulted in an increase of 1.30 ([0.09, 2.62] *p* = 0.034), 2.31 ([0.89, 3.57] *p* < 0.001), 2.67 ([1.70, 3.74] *p* < 0.001) and 2.70 years ([1.61, 3.74] *p* < 0.001) respectively, in the Bayesian A-ECG cardiac age. When adjusting for the AgeAccelPheno, the ACME for diabetes was 0.23 years ([0.01, 0.52] *p* = 0.028) and PM ≈18%, for high cholesterol 0.34 ([0.03, 0.74] *p* = 0.024) and PM ≈15%, and for any CV risk factor 0.17 ([0.01, 0.41] *p* = 0.048) and PM ≈ 6%. Similarly, AgeAccel Grim appears to mediate ≈19% of the relationship between diabetes and Bayesian A-ECG, and ≈7% of the hypertension-Bayesian A-ECG association. However, there was no significant ACMEs for AgeAccelHannum or AgeAccelHorvath.

**Table 3:** The associations between CV risk factors and the ECG-based cardiac ages and the role of DNAm AgeAccel as a mediator of these relationships

ECG-based cardiac age	Model	Effect type	Body Mass Index		Diabetes		High cholesterol		Hypertension		Cardiovascular disease		Any CV risk factor	
			*β* (95% CI)	*p* value	*β* (95% CI)	*p* value	*β* (95% CI)	*p* value	*β* (95% CI)	*p* value	*β* (95% CI)	*p* value	*β* (95% CI)	*p* value
Bayesian A-ECG age	M1	Total	0.32 (0.21, 0.44)	** < 2e−16**	1.30 (0.09, 2.62)	**0.034**	2.31 (0.89, 3.57	** < 2e−16**	2.67 (1.70, 3.74)	** < 2e−16**	1.81 (− 0.58, 4.27)	0.150	2.70 (1.61, 3.74)	** < 2e−16**
	M2 **(**AgeAccelHannum**)**	ACME	0.00 (0.00, 0.01)	0.940	− 0.01 (− 0.11, 0.09)	0.950	0.01 (− 0.09, 0.11)	0.910	− 0.01 (− 0.08, 0.09)	0.940	0.01 (− 0.11, 0.23)	0.820	− 0.02 (− 0.17, 0.11)	0.740
		ADE	0.32 (0.21, 0.44)	** < 2e−16**	1.31 (0.08, 2.64)	**0.036**	2.30 (0.88, 3.55)	** < 2e−16**	2.68 (1.70, 3.75)	** < 2e−16**	1.80 (− 0.60, 4.23)	0.150	2.72 (1.61, 3.74)	** < 2e−16**
	M3 (AgeAccelHorvath)	ACME	0.02 (− 0.01, 0.04)	0.19	0.02 (− 0.17, 0.22)	0.818	0.03 (− 0.23, 0.35)	0.850	0.12 (− 0.02, 0.38)	0.160	0.07 (− 0.10, 0.54)	0.540	0.15 (− 0.07, 0.45)	0.170
		ADE	0.30 (0.19, 0.43)	** < 2e−16**	1.28 (0.07, 2.62)	**0.036**	2.28 (0.91, 3.57)	** < 2e−16**	2.55 (1.60, 3.61)	** < 2e−16**	1.74 (− 0.70, 4.15)	0.160	2.55 (1.41, 3.59)	** < 2e−16**
	M4 (AgeAccelPheno)	ACME	0.02 (− 0.01, 0.06)	0.160	0.23 (0.01, 0.52)	**0.028**	0.34 (0.03, 0.74)	**0.026**	0.08 (− 0.07, 0.28)	0.290	0.26 (− 0.05, 0.88)	0.150	0.17 (0.01, 0.41)	**0.048**
		ADE	0.30 (0.21, 0.44)	** < 2e−16**	1.08 (− 0.16, 2.45)	0.098	1.97 (0.53, 3.21)	**0.002**	2.59 (1.62, 3.64)	** < 2e−16**	1.55 (− 0.81, 4.01)	0.150	2.53 (1.40, 3.61)	** < 2e−16**
	M5 (AgeAccelGrim)	ACME	0.02 (− 0.01, 0.05)	0.064	0.24 (0.01, 0.60)	**0.030**	0.34 (− 0.02, 0.74)	0.068	0.19 (0.00, 0.42)	**0.048**	0.33 (− 0.06, 0.91)	0.100	0.26 (− 0.04, 0.57)	0.092
		ADE	0.30 (0.18, 0.43)	** < 2e−16**	1.06 (− 0.19, 2.43)	0.118	1.97 (− 0.02, 0.74)	0.068	2.48 (1.50, 3.74)	**0.04**	1.48 (− 0.91, 3.92)	0.250	2.44 (1.28, 3.50)	** < 2e−16**
DNN ECG-based age	M1	Total	0.59 (0.37, 0.82)	** < 2e−16**	1.72 (− 0.93, 4.35)	0.200	2.71 (− 0.05, 5.49)	0.054	2.72 (0.43, 5.01)	**0.020**	4.16 (0.18, 8.69)	**0.040**	3.20 (0.84, 5.74)	**0.008**
	M2 **(**AgeAccelHannum**)**	ACME	0.00 (− 0.02, 0.02)	0.920	0.02 (− 0.16, 0.31)	0.850	0.01 (− 0.23, 0.28)	0.910	0.05 (− 0.11, 0.33)	0.560	− 0.10 (− 0.58, 0.43)	0.752	0.09 (− 0.11, 0.44)	0.500
		ADE	0.59 (0.37, 0.82)	** < 2e−16**	1.70 (− 0.92, 4.36)	0.200	2.70 (− 0.05, 5.49)	0.058	2.67 (0.33, 4.99)	**0.024**	4.26 (0.16, 8.68)	**0.042**	3.11 (0.76, 5.71)	**0.010**
	M3 (AgeAccelHorvath)	ACME	0.02 (− 0.02, 0.08)	0.280	0.04 (− 0.28, 0.36)	0.840	0.05 (− 0.48, 0.67)	0.860	0.23 (− 0.04, 0.72)	0.152	0.21 (0.30, 1.14)	0.524	0.31 (− 0.16, 0.80)	0.206
		ADE	0.57 (0.35, 0.80)	** < 2e−16**	1.68 (− 0.96, 4.34)	0.210	2.66 (− 0.03, 5.29)	0.054	2.49 (0.09 4.83)	**0.034**	3.95 (0.06, 8.47)	**0.046**	2.89 (0.49, 5.54)	**0.020**
	M4 (AgeAccelPheno)	ACME	0.03 (− 0.05, 0.11)	0.530	0.24 (− 0.15, 0.74)	0.270	0.36 (− 0.24, 1.14)	0.276	0.13 (− 0.12, 0.53)	0.370	0.51 (− 0.12, 1.61)	0.176	0.27 (− 0.08, 0.82)	0.140
		ADE	0.56 (0.34, 0.80)	** < 2e−16**	1.48 (− 0.93, 4.35)	0.250	2.35 (− 0.23, 5.05)	0.084	2.59 (0.25, 4.95)	**0.030**	2.65 (− 0.24, 8.01)	0.064	2.93 (0.50, 5.52)	**0.016**
	M5 (AgeAccelGrim)	ACME	0.04 (− 0.01, 0.11)	0.100	0.48 (0.02, 1.22)	**0.028**	0.76 (0.04, 1.71)	**0.036**	0.46 (0.01, 1.07)	**0.046**	0.69 (− 0.10, 1.97)	0.100	0.68 (− 0.04, 1.47)	0.064
		ADE	0.55 (0.33, 0.79)	** < 2e−16**	1.24 (− 1.36, 4.03)	0.342	1.95 (0.04, 1.71)	0.160	2.26 (− 0.05, 4.70)	0.060	3.47 (− 0.40, 7.85)	0.070	2.52 (− 0.08, 5.18)	0.056

All reported analyses consisted of generalized linear models with Gaussian distributions and identity links. Significant p-values are in bold

M1 aimed to evaluate the total effects, and was adjusted for chronological age, sex, SEP (as a binary variable: manual and non-manual), smoking status (as current smoker, ex-smoker and never-smoked), alcohol comsunption (as the average number of alcohol units drank per day), physical activity status (as active or inactive) and white cell counts (i.e., naïve and exhausted CD8 + T-lymphocytes, CD4 + T-lymphocytes, B-cells, natural killer cells, granulocytes, and monocytes). Compared to M1, M2 was in addition adjusted for AgeAccelHannum, M3 for AgeAccelHorvath, M4 for AgeAccelPheno and M5 for AgeAccelGrim

ACME = average causal mediation effect; ADE = average direct effect; M = model. Other abbreviations as in Table 2

A unit increase in BMI resulted in a 0.59 years ([0.37, 0.82] *p* < 0.001) increase in the DNN ECG-based cardiac age. Moreover, having hypertension, CVD or any CV risk factor resulted in an increase of 2.72 ([0.43, 5.01] *p* = 0.020), 4.16 ([0.18, 8.69] *p* = 0.040) or 3.20 years ([0.84, 5.74] *p* = 0.008) respectively, in the DNN ECG-based cardiac age. Although diabetes and high cholesterol were associated with higher DNN ECG-based cardiac ages, these were not statistically significant. When adjusting for AgeAccelGrim, the ACME for diabetes was 0.48 years ([0.02, 1.22] *p* = 0.028) and PM ≈37%, for high cholesterol 0.76 ([0.04, 1.71] *p* = 0.036) and PM ≈33%, and hypertension 0.46 ([0.01, 1.07] *p* = 0.046) and PM ≈ 17%. However, there were no significant ACMEs for AgeAccelHannum, AgeAccelHorvath, or AgeAccelPheno.

### DNAm as a mediator downstream of CV risk factors and upstream of A-ECG disease scores

In general, when considering the associations between CV risk factors and the A-ECG-based LVSD and LVER scores, only AgeAccelPheno and AgeAccelGrim appear to be mediators (Table 4). AgeAccelPheno appears to mediate ≈15% of the relationships between BMI and the two A-ECG-based risk scores, ≈28% of the association between diabetes and the LVSD score, and ≈17% of the BMI-LVER score association. Similarly, AgeAccelGrim emerged as a significant mediator for the associations between BMI, diabetes, high cholesterol and hypertension, and the two A-ECG-based risk scores (all p < 0.05 for ACMEs). Importantly, AgeAccelGrim mediates ≈40% of the relationships between diabetes, high cholesterol and hypertension, and LVSD; and 30% of their assocaition with LVER disease score.

**Table 4:** The association between CV risk factors and A-ECG disease scores and the role of DNAm AgeAccel as a mediator of these relationships

A-ECG disease scores	Model	Effect type	Body Mass Index		Diabetes		High cholesterol		Hypertension		Cardiovascular disease		Any CV risk factor	
			*β* (95% CI)	*p*-value	*β (*95% CI)	*p* value	*β* (95% CI)	*p*-value	*β* (95% CI)	*p* value	*β* (95% CI)	*p* value	*β* (95% CI)	*p* value
LVSD	M1	Total	− 0.06 (− 0.11, − 0.01)	**0.010**	− 0.25 (0.74, 0.17)	0.284	− 0.42 (− 0.92, 0.01)	0.058	− 0.28 (− 0.63, 0.06)	0.116	− 0.48 (− 1.29, 0.24)	0.184	− 0.26 (− 0.65, 0.08)	0.170
	M2 **(**AgeAccelHannum**)**	ACME	0.00 (− 0.00, 0.00)	0.950	− 0.00 (− 0.04, 0.02)	0.860	− 0.00 (− 0.04, 0.03)	0.882	− 0.01 (− 0.05, 0.02)	0.690	0.01 (− 0.06, 0.08)	0.900	− 0.01 (− 0.06, 0.03)	0.670
		ADE	− 0.06 (− 0.11, − 0.01)	**0.010**	− 0.25 (0.74, 0.18)	0.280	− 0.42 (− 0.92, 0.01)	0.058	− 0.27 (− 0.66, 0.07)	0.130	− 0.49 (− 1.29, 0.26)	0.180	− 0.25 (− 0.65, 0.11)	0.180
	M3 (AgeAccelHorvath)	ACME	− 0.00 (− 0.01, 0.00)	0.282	− 0.01 (− 0.07, 0.03)	0.820	− 0.01 (− 0.07, 0.08)	0.902	− 0.03 (− 0.09, 0.01)	0.140	− 0.02 (− 0.13, 0.06)	0.680	− 0.04 (− 0.13, 0.01)	0.170
		ADE	− 0.06 (− 0.11, − 0.01)	**0.024**	− 0.24 (− 0.74, 0.18)	0.290	− 0.41 (− 0.93, 0.01)	0.064	− 0.25 (− 0.63, 0.09)	0.160	− 0.46 (− 1.29, 0.30)	0.220	− 0.22 (− 0.62, 0.14)	0.250
	M4 (AgeAccelPheno)	ACME	− 0.01 (− 0.02, − 0.00)	**0.046**	− 0.07 (− 0.18, − 0.01)	**0.034**	− 0.09 (− 0.20, − 0.01)	**0.018**	− 0.03 (− 0.09, 0.03)	0.300	− 0.08 (− 0.26, 0.02)	0.140	− 0.06 (− 0.14, − 0.01)	**0.024**
		ADE	− 0.05 (− 0.10, 0.00)	0.056	− 0.18 (− 0.66, 0.24)	0.426	− 0.33 (− 0.84, 0.10)	0.140	− 0.25 (− 0.63, 0.07)	0.140	− 0.40 (− 1.21, 0.3(	0.300	− 0.20 (− 0.60, 0.15)	0.266
	M5 (AgeAccelGrim)	ACME	− 0.01 (− 0.03, − 0.01)	**0.016**	− 0.11 (− 0.26, − 0.01)	**0.014**	− 0.18 (− 0.33, − 0.06)	**0.002**	− 0.12 (− 0.24, − 0.03)	**0.006**	− 0.12 (− 0.32, 0.02)	0.094	− 0.19 (− 0.33, − 0.07)	** < 2e−16**
		ADE	− 0.05 (− 0.09, − 0.01)	**0.046**	− 0.14 (− 0.61, 0.27)	0.520	− 0.24 (− 0.72, 0.17)	0.252	− 0.16 (− 0.53, 0.16)	0.334	− 0.36 (− 1.11, 0.35)	0.314	− 0.07 (− 0.48, 0.28)	0.710
LVER	M1	Total	− 0.28 (− 0.48, − 0.09)	**0.004**	− 1.34 (− 3.25, 0.44)	0.120	− 1.76 (− 3.85, − 0.16)	**0.048**	− 1.21 (− 2.52, 0.10)	0.082	− 2.75 (− 5.82, − 0.23)	**0.036**	− 1.61 (− 2.95, − 0.41)	**0.004**
	M2 **(**AgeAccelHannum**)**	ACME	0.00 (− 0.01, 0.010	0.918	− 0.01 (− 0.17, 0.09)	0.820	− 0.00 (− 0.18, 0.11)	0.866	− 0.03 (− 0.21, 0.06)	0.598	0.05 (− 0.21, 0.31)	0.800	− 0.04 (− 0.25, 0.09)	0.544
		ADE	− 0.28 (− 0.48, − 0.09)	**0.002**	− 1.33 (− 3.25, 0.42)	0.120	− 0.18 (− 3.85, 0.14)	0.074	− 1.18 (− 2.49, 0.17)	0.090	− 2.80 (− 5.86, − 0.20)	**0.032**	− 1.57 (− 2.82, − 0.35)	**0.006**
	M3 (AgeAccelHorvath)	ACME	− 0.02 (− 0.05, 0.01)	0.174	− 0.02 (− 0.28, 0.13)	0.790	− 0.03 (− 0.28, 0.29)	0.884	− 0.13 (− 0.38, 0.03)	0.124	− 0.10 (− 0.47, 0.24)	0.650	− 0.18 (− 0.47, 0.03)	0.106
		ADE	− 0.26 (− 0.47, − 0.09)	**0.006**	− 1.32 (− 3.16, 0.47)	0.130	− 1.73 (− 3.84, 0.18)	0.084	− 1.08 (− 2.45, 0.25)	0.118	− 2.65 (− 5.89, − 0.01)	**0.049**	− 1.43 (− 2.75, − 0.22)	**0.008**
	M4 (AgeAccelPheno)	ACME	− 0.04 (− 0.09, − 0.00)	**0.049**	− 0.23 (− 0.58, − 0.01)	**0.042**	− 0.29 (− 0.73, 0.01)	0.054	− 0.11 (− 0.33, 0.10)	0.300	− 0.26 (− 0.88, 0.09)	0.172	− 0.22 (− 0.50, − 0.02)	**0.030**
		ADE	− 0.24 (− 0.45, − 0.04)	**0.018**	− 1.11 (− 3.0, 0.71)	0.214	− 1.47 (− 3.67, 0.48)	0.152	− 1.10 (− 2.41, 0.25)	0.096	− 2.49 (− 5.70, 0.36)	0.080	− 1.39 (− 2.68, − 0.26)	**0.012**
	M5 (AgeAccelGrim)	ACME	− 0.04 (− 0.09, − 0.01)	**0.010**	− 0.36 (− 0.87, − 0.02)	**0.018**	− 0.62 (− 1.20, − 0.15)	**0.008**	− 0.37 (− 0.77, − 0.07)	**0.012**	− 0.38 (− 1.12, 0.06)	0.110	− 0.56 (− 1.09, − 0.10)	**0.004**
		ADE	− 0.24 (− 0.42, − 0.05)	**0.008**	− 0.98 (− 2.84, 0.68)	0.252	− 1.14 (− 3.15, 0.68)	0.244	− 0.84 (− 2.12, 0.51)	0.188	− 2.37 (− 5.22, 0.20)	0.068	− 1.05 (− 2.37, 0.13)	0.082

All reported analyses consisted of generalized linear models with Gaussian distributions and identity links. Significant p-values are in bold

M1 aimed to evaluate the total effects, and was adjusted for chronological age, sex, SEP (as a binary variable: manual and non-manual), smoking status (as current smoker, ex-smoker and never-smoked), alcohol consumption (as the average number of alcohol units drank per day), physical activity status (as active or inactive) and white cell counts (i.e., naïve and exhausted CD8+ T-lymphocytes, CD4+ T-lymphocytes, B-cells, natural killer cells, granulocytes, and monocytes). Compared to M1, M2 was in addition adjusted for AgeAccelHannum, M3 for AgeAccelHorvath, M4 for AgeAccelPheno and M5 for AgeAccelGrim. Abbreviations as in Table 3

### Sensitivity analysis

The ρ at which ACME = 0 is presented in Additional file 1: Supplementary Table S1. In general, even small deviations from the SI assumption can reverse the sign of ACME.

## Discussion

In this cross-sectional analysis, we show that the association between CV risk factors and ECG-based cardiac ages and disease scores could be partly mediated by the 2nd generation DNAm AgeAccel biomarkers. AgeAccelPheno and AgeAccelGrim appear to mediate the relationships between most CV risk factors and the Bayesian A-ECG cardiac age, and the LVSD and LVER disease scores AgeAccelGrim also appears to mediate the relationship between most CV risk factors and the DNN ECG-based cardiac age. However, this mediation appears to account for ≈10–40% of the total effects. AgeAccelHannum and AgeAccelHorvath appear to have a limited role.

Although epigenetics is a broad topic, our understanding revolves mainly on DNAm at CpG sites which are concentrated in the promoter regions of the genes albeit sparse in other parts of the genome. In general, promoter methylation at CpG sites can lead to gene silencing, whilst unmethylated promoters remain transcriptionally active. Previous epigenome-wide association studies have highlighted DNAm profiles associated with cardiometabolic risk factors (e.g., BMI [23], diabetes [24], high cholesterol [25], and hypertension [26]). In this study we show that DNAm AgeAccel derived from CpG methylation at specific sites in different tissues (especially blood) is a partial mediator downstream of the CV risk factors but upstream of the A-ECG phenotypes. This reinforces the theory that CV risk factors can lead to dynamic DNA changes with potentially adverse long-term cardiac phenotypic sequelae. Indeed, DNMT-i showed promising results in pre-clinical trials as they reduced pathological hypertension-related myocardial hypertrophy [28] and fibrosis [29], and ameliorated atherosclerosis [30]. Interestingly, aspirin which has a proven benefit especially in the secondary prevention of CVDs, might also exert some of its effects by acting as a DNMT-i [48]. In general, DNMT-i have a reversible effect suggesting that their safety, efficacy, and effectiveness in protecting against pathological age-related remodeling in human clinical trials might be possible to explore [49].

**Fig. 2:**
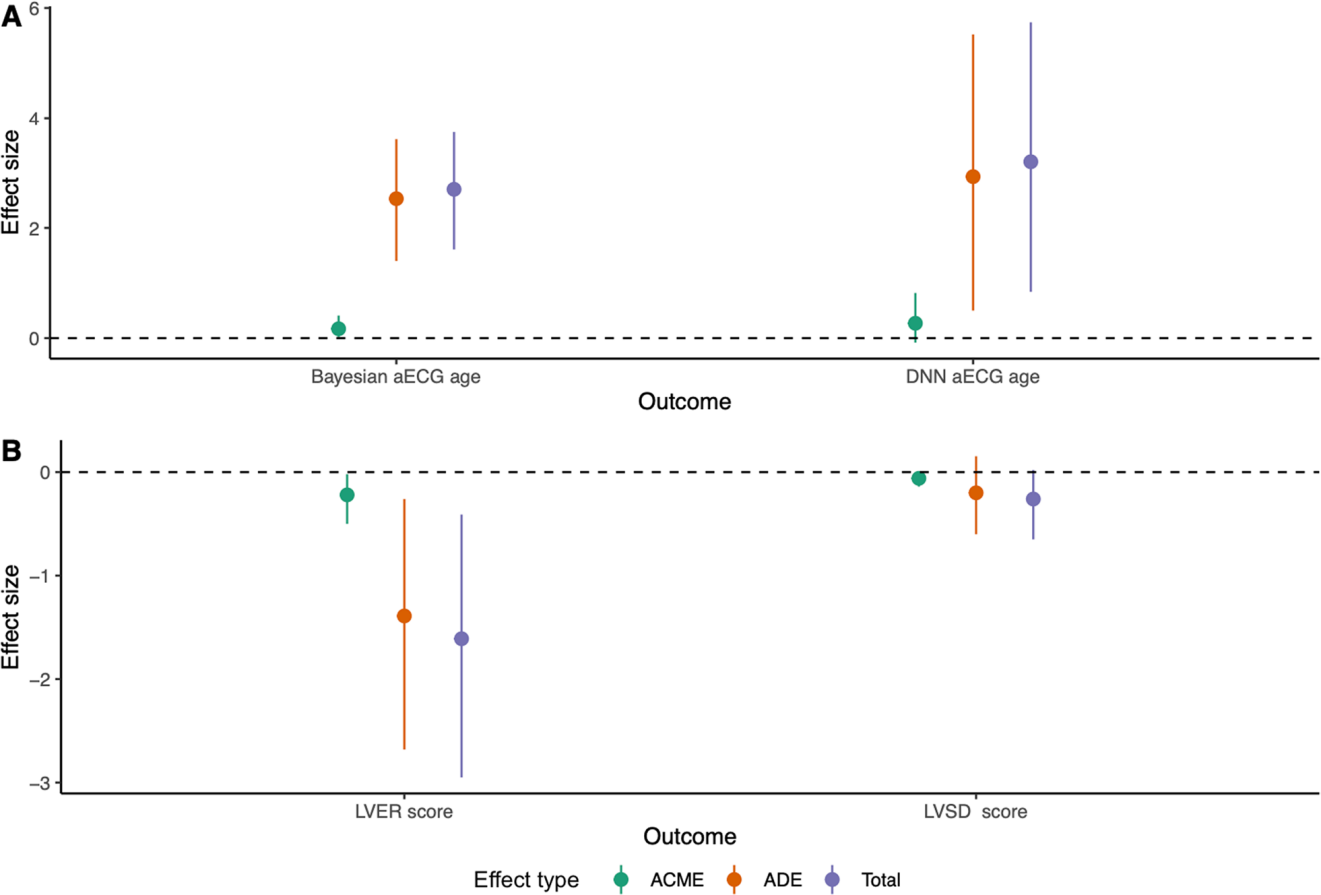
**The role of DNAm AgeAccelPheno in mediating the relationship between any CV risk factor and the ECG-based outcomes.** We defined the presence of any CV risk factor as participants having at least one out of: diabetes, high cholesterol, hypertension, CVD, or a BMI > 30. On average, DNAm AgeAccelPheno mediates 10–20% of the relationships between any CV risk factor and the ECG-based cardiac ages (panel **A**) and disease scores (panel **B**). Either  a more positive ECG-based cardiac age or  a more negative ECG-based disease score represent a worse phenotype. Abbreviations: aECG = advanced electrocardiography; ACME = average causal mediation effect; ADE = average direct effect; DNN = deep neural network; LVER = left ventricular electrical remodelling; LVSD = left ventricular systolic dysfunction. Other abbreviations as in Fig. 1

Although the 1st generation DNAm ages appeared to be only weak predictors of CVDs [22], the 2nd generation DNAm ages  incorporating clinical and physiological prognostic methylation biomarkers were intended to act as better markers of healthspan (DNAm PhenoAge) and lifespan (DNAm GrimAge). Our findings suggest that only the 2nd generation DNAm AgeAccel metrics could act as mediators of the association between the CV risk factors and the ECG-based cardiac outcomes. DNAm GrimAge incorporates blood-based biomarkers related to extracellular matrix (ECM) remodeling (e.g., epidermal growth factor-containing fibulin-like ECM protein, plasminogen activator inhibitor 1, tissue inhibitor metalloprotease 1 etc.) [7]. As LVSD and LVER are characterized by extensive remodeling, it is not surprising that DNAm AgeAccelGrim emerged as a significant mediator downstream of CV risk factors but upstream of these A-ECG disease scores. In contrast, DNAmPheno encompasses CpG sites related to immune system (e.g., white blood cell count), inflammation (e.g., C-reactive protein) and metabolism (e.g., glucose). Inflammaging [50] and immunosenescence [51] have both been associated with faster cardiac aging and increased susceptibility to CVDs as they augment endothelial damage, impair tissue repair, and promote insulin resistance and atherosclerosis [52]. Given this strong relationship between immunity and metabolism, and cardiac ageing, DNAm AgeAccelPheno emerged as a consistent mediator in the association between CV risk factors and A-ECG ages and disease scores in our study (Fig. 2).

According to our results, only 10–40% of the total effects of the association between CV risk factors and A-ECG phenotypes are mediated by DNAm AgeAccel. However, the proportion mediated as a numerical quantity provides limited insights into the relative importance of the DNAm pathway. Whether DNAm has only a limited impact or whether it is a major stem which sets into motion a raft of cascading pathological cardiac ageing pathways, remains to be further elucidated. Moreover, the directionality of effect could not be firmly deduced using the analyses provided in this study. While it is theoretically possible that a more advanced cardiac age leads to a higher DNAm age, it is more biologically plausible that CV risk factors induce physiological stress driving DNAm. The advent of the International Human Epigenome Consortium promises to provide novel insights into the epigenetic changes most strongly associated with cardiac ageing [53]. As CpG methylation is closely linked to gene transcription, the transcriptome and proteome, now measurable via high-throughput RNA sequencing and shotgun proteomics, respectively, will bolster our understanding of the mechanisms downstream of DNAm but upstream of the cardiac ageing phenotypes.

### Strengths and limitations

NSHD was representative of a British-born population at the time of participant recruitment. The implicit age homogeneity of the birth cohort participants was another strength of the study, as it enables age-matching across analyses meaning that age-related confounding was minimized. In addition, participants were exposed to similar secular trends and risk factors, and similar access to diagnostic technologies and treatment facilities over time, which minimizes the bias of environmental factors.

An important limitation is that only participants who had DNAm and analyzable 12-lead ECGs which were collected as part of two different separate sub-studies were included in this study. Recently, the updated version of DNAm GrimAge was published, but this was not available at the time of the current analysis [54]. As this study was retrospectively designed, selection bias may have influenced the observed associations. Moreover, the study has all the limitations inherent to cross-sectional studies (e.g., antecedent-consequent bias, susceptibility to transient effects etc.). In addition, the limited sample size meant that we were underpowered to significantly detect certain associations, with statistically significant ACMEs but not total effects found in some analyses. In that case, although we could not claim an association between the CV risk factor and the corresponding ECG-based outcome, if indeed there is one it is probably being mediated by the DNAm variable. Although sex-specific differences between DNAm and CV health have been reported, this study was underpowered for sex-stratified analyses, so they were not pursued. Moreover, we assumed that no exposure-mediator or mediator-outcome interactions exists. Whist mediation frameworks taking into account interactions exists (e.g., VanderWeel’s four-way decomposition [55]), they are more suitable for studies with larger sample sizes. Similarly, the results are sensitive to the violation of the SI assumption (Additional file 1: Supplementary Table S1) and the existence of unexplored confounding cannot be excluded although we adjusted for chronological age, sex, SEP, smoking, alcohol, and physical activity. Lastly, repeating the analyses using the sub-components of the DNAm ages would have provided a more comprehensive understanding of the association between CV risk factors and the ECG-outcomes, and this represents our plans for future work.

## Conclusion

By the age of 60, individuals with accelerated DNA methylation appear to have older, weaker, and more electrically impaired hearts. The harmful effects of CV risk factors on cardiac age and health, appear to be partially mediated by the 2nd generation DNA methylation age biomarkers. This highlights the need for more research into the potentially cardioprotective roles of selective DNA methyltransferases modulators.

### Supplementary Information

**Additional file 1:** Sensitivity analysis for the mediation analyses.

## Data Availability

NSHD data are available from: https://www.nshd.mrc.ac.uk/data. The mediation analysis code template used can be accessed here: https://cran.r-project.org/web/packages/mediation/mediation.pdf.
