# Supplementary material for: Accelerated DNA methylation age plays a role in the impact of cardiovascular risk factors on the human heart
Source: Clin Epigenetics. 2023 Oct 18;15:164. doi: 10.1186/s13148-023-01576-9 (PMC10583368; doi:10.1186/s13148-023-01576-9)
Supplement: Supplementary file 1 — Additional file 1: Sensitivity analysis for the mediation analyses. [file 13148_2023_1576_MOESM1_ESM.docx]

**ADDITIONAL FILE 1 FOR**

**Accelerated DNA methylation age plays a role in the impact of cardiovascular risk factors on the human heart**

Constantin-Cristian Topriceanu ^1,2,3^, Eesha Dev ^4^, Mahmood Ahmad^5^, Rebecca Hughes^2,3^, Hunain Shiwani^2,3^, Matthew Webber^1,2^, Kenan Direk^1^, Andrew Wong^1^, Martin Ugander^6^, James C Moon ^2,3^, Alun D Hughes^1,2^, Jane Maddock^1,2^, Todd T. Schlegel^7,8^, Gabriella Captur ^1,2,5^

**Author Affiliations:**

1. UCL MRC Unit for Lifelong Health and Ageing, University College London, London, UK
2. UCL Institute of Cardiovascular Science, University College London, London, UK
3. Cardiac MRI Unit, Barts Heart Centre, West Smithfield, London, UK
4. UCL Medical School, Gower Street, London, UK
5. The Royal Free Hospital, Centre for Inherited Heart Muscle Conditions, Cardiology Department, Pond Street, Hampstead, London, UK
6. Kolling InstituteRoyal North Shore Hospital, and Charles Perkins CentreFaculty of Medicine and Health, University of Sydney, Sydney Australia.
7. Department of Clinical Physiology, Karolinska University Hospital, and Karolinska Institutet, Stockholm, Sweden.
8. Nicollier-Schlegel SARL, Trélex, Switzerland

**Corresponding author:**

Gabriella Captur

Consultant Cardiologist in Inherited Heart Muscle Conditions, Senior Clinical Lecturer,

Institute of Cardiovascular Science, University College London, London WC1E 6BT, UK

E-mail: [gabriella.captur@ucl.ac.uk](mailto:gabriella.captur@ucl.ac.uk), Phone No: +44 2074600595

**Supplementary Table S1. Sensitivity analysis for the mediation analyses.**

| **ECG-based age** | **Model** | **BMI** | **Diabetes** | **High Cholesterol** | **Hypertension** | **CVD** | **Any CV risk factor** |
| --- | --- | --- | --- | --- | --- | --- | --- |
| Bayesian A-ECG age | AgeAccelHannum | 0.0 | 0.0 | 0.0 | 0.0 | 0.0 | 0.0 |
|  | AgeAccelHorvath | 0.1 | 0.1 | 0.1 | 0.1 | 0.0 | 0.1 |
|  | AgeAccelPheno | 0.1 | 0.1 | 0.1 | 0.1 | 0.1 | 0.1 |
|  | AgeAccelGrim | 0.0 | 0.0 | 0.0 | 0.0 | 0.0 | 0.0 |
| DNN  ECG-based age | AgeAccelHannum | 0.1 | 0.0 | 0.0 | 0.0 | 0.1 | 0.0 |
|  | AgeAccelHorvath | 0.0 | 0.0 | 0.0 | 0.0 | 0.0 | 0.0 |
|  | AgeAccelPheno | 0.0 | 0.1 | 0.1 | 0.1 | 0.1 | 0.1 |
|  | AgeAccelGrim | 0.1 | 0.1 | 0.1 | 0.1 | 0.1 | 0.1 |
| LVSD | AgeAccelHannum | -0.1 | 0.0 | 0.0 | -0.1 | -0.1 | -0.1 |
|  | AgeAccelHorvath | 0.0 | -0.1 | -0.1 | -0.1 | 0.0 | -0.1 |
|  | AgeAccelPheno | -0.1 | -0.1 | -0.1 | -0.1 | -0.1 | -0.1 |
|  | AgeAccelGrim | -0.1 | -0.1 | -0.1 | -0.1 | -0.1 | -0.1 |
| LVER | AgeAccelHannum | -0.1 | -0.1 | -0.1 | -0.1 | -0.1 | -0.1 |
|  | AgeAccelHorvath | -0.1 | -0.1 | -0.1 | -0.1 | 0.0 | -0.1 |
|  | AgeAccelPheno | -0.1 | -0.1 | -0.1 | -0.1 | -0.1 | -0.1 |
|  | AgeAccelGrim | -0.1 | -0.1 | -0.1 | -0.1 | -0.1 | -0.1 |

We previously explored to what extent DNAm AgeAccel is mediating the associations between the CV risk factors and A-ECG cardiac ages and disease scores. To investigate the robustness of our results, we used the Baron-Kenny procedure to calculate the ρ between the residuals of the mediator and the regression outcome from the linear structural equation models. The magnitude of ρ essentially represents the deviation from the SI assumption about the mediator. The higher the ρ, the higher the magnitude of the correlation required to reverse the sign of ACME, the more robust are the results in the face of the SI violation.

As we judge the robustness of the results by the magnitude of ρ required to reverse the sign of ACME (rather than ADE), we report the ρ at which ACME=0.

*ρ =correlation coefficient; ACME = average causal mediation effect; ADE = average direct effect; A-ECG = advanced electrocardiography; AgeAccel = age acceleration; BMI = body mass index; CV = cardiovascular, CVD = cardiovascular disease; DNAm = DNA methylation; DNN = deep neural network; LVER = left ventricular electrical remodeling; LVSD = left ventricular systolic dysfunction; SI = sequential ignorability.*
